# Empowering menopausal women to adopt climate-adaptive health behaviors and reduce climate-sensitive health outcomes: an IMB model-based quasi-experimental study

**DOI:** 10.3389/fpubh.2026.1908834

**Published:** 2026-07-24

**Authors:** Ola Abdel-Wahab Afifi Araby, Nesma Abd-Elaziz Ibrahim El Desoky, Abdelaziz Hendy, Taghreed Rasheed Aljaloud, Sahar Shafeek Mohamed Afify

**Affiliations:** 1 Obstetrics and Gynecology Nursing, Faculty of Nursing, Benha University, Benha, Egypt; 2 Department of Maternity and Child Health Nursing, College of Nursing, Qassim University, Buraydah, Saudi Arabia; 3 Nursing Services Department, Qassim University Medical City, Buraydah, Saudi Arabia

**Keywords:** climate change, health behavior, health education, health status, menopause, motivation, nursing care, women’s health

## Abstract

**Background:**

Climate change poses increasing health risks for menopausal women. Therefore, evidence-based behavioral models are needed to enhance their knowledge, motivation, and skills to support climate-resilient health behaviors.

**Aim:**

This study aimed to examine the effect of the Information–Motivation–Behavioral Skills (IMB) model on empowering menopausal women and improving perceived climate-sensitive menopausal health outcomes.

**Methods:**

A quasi-experimental one-group time-series design (pre-intervention, post-intervention, and follow-up) was used. The study was conducted at the administrative building of Benha University Hospital. A purposive sample of 58 menopausal women was recruited according to inclusion criteria. Data were collected using four tools: a structured socio-demographic and environmental health awareness questionnaire, an environmental motivation scale, a climate change health protection behaviors scale, and a perceived climate-sensitive menopausal health outcomes questionnaire. Repeated-measures ANOVA was used for analysis, and effect sizes were reported using partial eta squared (η^2^p). Assumptions of normality and sphericity were assessed prior to analysis.

**Results:**

The findings revealed statistically significant improvements in menopausal women’s environmental awareness, motivation, climate change health protection behaviors, and perceived menopausal health outcomes across pre-intervention, post-intervention, and follow-up phases (*p* < 0.001). Improvements were sustained at follow-up, indicating positive changes over time. Effect sizes indicated small- to large-magnitude improvements across outcomes.

**Conclusion:**

The IMB model was associated with improvements in menopausal women’s awareness, motivation, and protective behaviors, as well as improvements in perceived health outcomes over time. However, due to the quasi-experimental design and absence of a control group, causal inferences cannot be established. The findings support the potential value of integrating the IMB model into nursing and multidisciplinary health education strategies, particularly in climate-vulnerable populations of menopausal women.

## Introduction

Recently, climate change has emerged as a critical public health challenge driving long-term alterations in temperature, precipitation, wind patterns, and other elements of the Earth’s climate system, primarily driven by anthropogenic greenhouse gas emissions (1–3). These environmental changes significantly affect human health through increased heat exposure, air pollution, and extreme weather events, producing complex hazards to human well-being (4, 5).

Evidence indicates that for every 1 °C rise in global temperatures, heat-related morbidity and mortality rise by 18–35%, with older women among the most affected group (6). Women are disproportionately vulnerable to climate-sensitive health risks, particularly menopausal women, owing to physiological vulnerabilities and altered thermoregulation (7). These vulnerabilities are further exacerbated in resource-limited settings by limited access to health services, gendered roles, and cultural norms that restrict adaptive options (8–10).

Menopause, which typically occurs between 45 and 55 years of age and is characterized by the cessation of menstruation for 12 consecutive months, is accompanied by declining ovarian follicular function, vasomotor symptoms (hot flushes/night sweats), sleep disturbance, and mood swings (11, 12). Scientific evidence indicates that climate change, higher temperatures, and humidity correlate with increased vasomotor symptom severity (13–16). Beyond these manifestations, it also compromises sleep quality, cardiovascular well-being, bone health, and mental resilience among menopausal women (7, 17). Menopause represents a golden moment for implementing virtuous behaviors that will benefit women’s health (18). Globally, the number of women transitioning through menopause is rising, and projections suggest that by 2030 approximately 1.2 billion women will be postmenopausal (19). Moreover, menopausal symptoms such as vasomotor instability affect up to 80% of women and may persist for 4–10 years after menopause (20).

Targeted nursing interventions are therefore needed to equip menopausal women with the knowledge, motivation, and practical skills required to implement adaptive health behaviors, including adequate fluid intake, personal cooling strategies, appropriate clothing, hygiene practices, and sleep regulation. Such behaviors can strengthen physiological resilience and mitigate climate-sensitive health outcomes during menopause. The urgency of this agenda is underscored by demographic projections indicating rapid global growth in the number of postmenopausal women, alongside the high prevalence and prolonged duration of menopausal symptoms (7).

The Information–Motivation–Behavioral Skills (IMB) model, originally developed by Fisher and Fisher (21), offers a robust theoretical framework for understanding and promoting health-related behavior (22, 23). According to the IMB model, behavioral change is driven by the interaction of three essential components: accurate and relevant health information, personal and social motivation, and the behavioral skills necessary to perform the desired actions to translate knowledge and motivation into practice (24).

The nursing discipline plays a critical role in advancing the United Nations Sustainable Development Goals (SDGs) (25). By integrating the IMB model, nursing can play a pivotal role in empowering menopausal women to proactively reduce climate-sensitive health outcomes while advancing sustainable health practices and public health goals, reinforcing the role of nursing as a cornerstone in achieving comprehensive and sustainable health outcomes worldwide (26).

Despite the growing global focus on women’s climate health, limited research has examined behavioral interventions targeting menopausal women in low- and middle-income settings such as Egypt. To address this gap, the present study implements a nursing intervention based on the Information-Motivation-Behavioral Skills (IMB) model to enhance climate-resilient health behaviors and improve perceived health outcomes related to climate sensitivity among menopausal women.

## Aim of the research

This study aimed to investigate the effect of utilizing the information-motivation-behavioral skills model to empower menopausal women to target perceived climate-sensitive menopausal health outcomes.

### Research hypotheses

H1: Menopausal women will have improved environmental health awareness related to climate change after the implementation of the information-motivation-behavioral skills model compared to before. H2: Menopausal women will have greater intrinsic and extrinsic environmental motivation after the implementation of the information-motivation-behavioral skills model than before. H3: Menopausal women will be more engaged in climate change health protection behaviors after the implementation of the information-motivation-behavioral skills model than before. H4: Menopausal women will have improved perceived climate-sensitive menopausal health outcomes after the implementation of the information-motivation-behavioral skills model.

H0: There will be no difference in information, motivation, and behavioral skills related to environmental health awareness and climate change among menopausal women after the application of the Information–Motivation–Behavioral Skills (IMB) model compared to before its application.

## Subjects and method

### Research design

A quasi-experimental research design (one group, time series: pre, post, and follow-up design) was used to fulfill the aim of the research. Quasi-experimental studies involve manipulation of independent variables to observe their effect on dependent variables, referred to as pre-post-intervention designs, and are often used to explore causal relationships. Quasi-experiments may lack randomization and/or control group characteristics of true experiments (27).

### Research setting

The research was conducted at the administrative building of Benha University Hospitals. It is one of the main buildings of the Benha University Hospital. The building comprises groups of clerks of different ages and genders working from 9 a.m. to 2 p.m., except on Fridays and official holidays. It consists of six floors that host various departments, including public services, administrative offices, financial units, quality assurance, infection control, and information technology serving patients, staff, and healthcare providers.

### Sampling

#### Sample type, size, and criteria

A purposive sample of 58 menopausal women was selected *according to specified inclusion criteria*: married menopausal women aged 45 to 55 years; menopausal women who had reached natural menopause without other pathological or surgical causes; women free from chronic physical and mental disorders; women who had not participated in a similar educational or behavioral intervention related to environmental or climate change awareness before; and women who could read and write.

The sample size was determined *a priori* using a power-based calculation for a one-group pre–post quasi-experimental design. Assuming a 95% confidence level, 80% statistical power, and an expected moderate effect size (Cohen’s d = 0.5) derived from previous similar IMB-based intervention studies, the minimum required sample size was estimated. Accordingly, a final sample of 58 participants was recruited to ensure adequate statistical power. The effect size assumption was guided by Cohen’s criteria for small, medium, and large effects in behavioral and health research (28). The sample size estimation was performed in accordance with standard methodological guidance for health intervention studies (29).

### Tools of data collection

Four tools were utilized for data collection; all tools were translated into Arabic, the mother tongue of the research participants.

Tool (I): A structured self-administered questionnaire: It was constructed by researchers after reviewing related literature and was written in Arabic in the form of closed-ended questions. It was included in two parts.

Part (1): Personal characteristics of women: This section consisted of six items, which were age, duration since menopause, residence, level of education, occupation, and monthly income.

Tool II: Environmental Motivation Scale (EMS): This was adopted from Çiçek Şentürk and Selvi (30) and used to assess factors that motivate women to adopt healthy behaviors towards the environment to reduce climate change and deal with its effects. The scale consists of 16 items divided into two factors: *the first factor is “Intrinsic Motivation,”* and the items were 1st, 3rd, 7th, 5th, 11th, 18th, 19th, 20th, 22nd, and 29th (I believe that the natural habitats of relics must be preserved; I do not want the natural habitats of plants to be destroyed; I do not want habitats of animals to be harmed; I would like to know the damages of polluted waters to sea creatures, etc.). *The second factor, “Extrinsic Motivation,”* included items 2nd, 4th, 10th, 15th, 24th, and 27th (I can investigate environmental problems to show that I am better than others; I would like to participate in environmental activities (planting trees, etc.) to show that I am better than others; I am not polluting the environment for my family to appreciate me; I want to join the environmental protection club at the school to make good friendships with others…etc.).

Scoring system: The scale was prepared as a three-point Likert scale. The response is graded from 2 for “agree” or 1 for “somewhat” or 0 for “disagree.” The lowest score that could be taken on this scale was 0, and the maximum score was 32, with a higher score indicating more motivation. *The total motivation score was categorized as follows:* High motivation: ˃ High motivation is defined as scoring 75% or more of the total score (25–32 points); moderate motivation is defined as scoring between 50 and 75% of the total score (16–24 points); and low motivation is defined as scoring less than 50% of the total score (0–15 points).

Tool III: Climate Change Health Protection Behaviors Scale (CCHPB): This was adopted from DeVellis and Thorpe (31) and Kurt et al. (32). The scale is designed to evaluate the health protection behaviors of people in situations that may arise as a result of climate change and can directly impact health and increase disease risk. It is a 28-item scale consisting of four factors. *Factor 1* pertains to obtaining accurate information about global climate change to protect health (items 1–5) (e.g., I follow accurate information frequently, I learn by asking health professionals, I learn from books, etc.). *Factor 2* relates to health behaviors to be implemented when faced with situations caused by climate change (items 6–15), such as seeking shelter indoors, eating more fruits and vegetables, eating more foods that strengthen my immune system, increasing my fluid intake, etc. *Factor 3* includes health behaviors to be performed after the situations caused by climate change have passed (items 16–22) (e.g., eating less spicy and hot food, increasing my exercise time, increasing my sleep time, and paying more attention to my personal hygiene). *Factor 4* included health behaviors to mitigate climate change (items 23–28), such as preferring walking or cycling instead of public transportation, preferring to use stairs instead of elevators, and separating waste such as paper, glass, and household waste and ensuring that it is disposed of. Score system, with each item rated on a three-point Likert scale. Responses were graded as follows: strongly agree (4), mostly agree (3), moderately agree (2), partly agree (1), and disagree (0). The lowest and highest possible scores were 0 and 112, respectively, with higher scores indicating better health protection behavior. *The overall score for health protection behaviors was categorized as follows:* Satisfactory behaviors: > 60% of the total score (68–112 score) and unsatisfactory behaviors: ≤ 60% of the total score (0–67).

Tool IV: The Perceived Climate-Sensitive Menopausal Health Outcomes Questionnaire was developed by the researchers after reviewing related literature (33–35) to assess menopausal women’s perceptions of climate-sensitive health outcomes based on the severity of their complaints. The questionnaire was composed of 18 items (symptoms or complaints) and categorized under three domains: *physical outcomes*, including hot flushes, night sweats, heart palpitations, recurrent infections, sleeping disturbances, muscle and joint pain, lack of energy, physical discomfort, and exhaustion. *Psychological outcomes* included depressive mood, irritability, anxiety, stress, mood swings, and lack of concentration. *Relationship and activity-related outcomes* included lack of sexual desire, impaired social relations, limited daily activities, and decreased work productivity.

Scoring system: Each item in the scale is scored on a 5-point Likert scale, starting from 0 (no symptoms) to 4 (very severe symptoms). The total score was obtained by adding all the points of each item and ranged between 0 (asymptomatic) and 72 (severe symptoms). Higher scores indicate worsening of subjectively perceived symptoms. *Summation scores for perceived climate-sensitive menopausal health outcomes were classified as follows:* excellent health outcomes = 0–18 score, satisfactory health outcomes = 19–36 score, average health outcomes = 37–54 score, and poor health outcomes = 55–72.

### Validity and reliability of the tools

The data collection tools were reviewed by a panel of three experts in obstetrics and gynecological nursing to ensure their validity for comprehensiveness, accuracy, and relevance. No modifications were made. From the experts’ perspective, the tools were considered valid. The reliability of the tools was assessed using Cronbach’s alpha coefficient test. The Environmental Motivation Scale demonstrated Cronbach’s alpha values of 0.76 for intrinsic motivation and 0.71 for extrinsic motivation, indicating satisfactory reliability for both the subscales. The Climate Change Health Protection Behaviors Scale (CCHPB) showed a Cronbach’s alpha of 0.874 for the total scale, reflecting excellent internal consistency across dimensions. Finally, the climate change-sensitive health outcome scale had a Cronbach’s alpha of 0.88, indicating high internal reliability.

### Field work

The field work was conducted over a six-month period from the beginning of April 2025 to the end of September 2025. The researchers visited the study setting 2 days per week (Sundays and Thursdays) from 9:00 a.m. to 1:00 p.m. At the beginning of each interview, participants were greeted and informed about the purpose of the study. After obtaining written informed consent, data collection was carried out using a structured self-administered questionnaire. The questionnaire included socio-demographic characteristics and validated tools to assess environmental health awareness, environmental motivation (intrinsic and extrinsic), climate change-related health protection behaviors, and perceived climate-sensitive menopausal health outcomes (including physical, psychological, relational, and activity-related domains).

Based on the findings of the baseline assessment and an extensive review of the relevant literature, the researchers identified the educational needs of menopausal women. Accordingly, an educational booklet in Arabic, enriched with colorful illustrations, was developed to enhance environmental awareness, strengthen intrinsic and extrinsic motivation, and improve climate-related health protection behaviors, with the ultimate aim of improving perceived menopausal health outcomes.

The intervention was implemented in a designated room equipped with seating arrangements, a data show projector, and supporting educational materials. Participants were divided into eight subgroups, each consisting of 7–8 women. Each subgroup received five structured educational sessions based on the Information–Motivation–Behavioral Skills (IMB) model. Sessions were conducted weekly and lasted 30–45 min each.

The intervention focused on three core components: information provision (menopause, climate-related health risks, and protective practices), motivation enhancement (intrinsic and extrinsic motivational factors), and behavioral skills training (hydration, nutrition, energy conservation, waste management, and coping strategies for climate-sensitive menopausal symptoms such as hot flushes, sleep disturbances, fatigue, and mood changes). Interactive teaching methods were used, including group discussion, demonstrations, role-playing, and individualized action planning. Each participant received the educational booklet at the beginning of the program to support continuous learning.

Post-intervention assessment was conducted 1 month after completion of the intervention program, using the same data collection tools (Tool I, Part 2, and Tools II, III, and IV). In addition, a follow-up assessment was performed 2 months after the post-intervention evaluation to assess the sustainability of the intervention effects over time.

### Statistical analysis

Data were analyzed using IBM SPSS Statistics (version 26). Descriptive statistics were presented as means ± standard deviations (SD) for continuous variables and frequencies and percentages for categorical variables. Prior to inferential analysis, data distribution was assessed for normality using the Shapiro–Wilk test and the Kolmogorov–Smirnov test for overall assessment. The results indicated acceptable normality for the studied variables, as follows: Shapiro–Wilk test values ranged from W = 0.955 to 0.983 with corresponding *p*-values ranging from 0.028 to 0.203, while Kolmogorov–Smirnov test values ranged from D = 0.068 to 0.102 with p-values ranging from 0.180 to 0.200. In addition, normality was visually inspected using histograms and Q–Q plots and statistically evaluated using skewness and kurtosis values. Skewness values ranged between −0.58 and 0.67, while kurtosis values ranged between −0.90 and 1.12, indicating acceptable symmetry and distributional shape within the recommended threshold of ±1.96. Variables were considered normally distributed when *p* > 0.05 in Shapiro–Wilk/Kolmogorov–Smirnov tests and when skewness and kurtosis values fell within the acceptable range.

Accordingly, repeated-measures ANOVA was used to compare mean differences across the three time points (pre-intervention, post-intervention, and follow-up) for normally distributed variables. For non-normally distributed data, appropriate non-parametric alternatives (Friedman test) were considered; however, all primary outcome variables met parametric assumptions. Mauchly’s test indicated that the assumption of sphericity was violated for the repeated-measures factor (W = 0.82, χ^2^(2) = 12.41, *p* = 0.002); therefore, Greenhouse–Geisser corrections (*ε* = 0.78) were applied to adjust the degrees of freedom in the repeated-measures ANOVA. Post-hoc pairwise comparisons with Bonferroni adjustment were performed to identify specific differences between time points. To control for type I error due to multiple comparisons, Bonferroni correction was applied where appropriate. The Pearson correlation coefficient (r) was used to examine the relationships between the total scores of the study variables.

Effect sizes were calculated using partial eta squared (η^2^p) and interpreted according to Cohen’s guidelines (0.01 = small, 0.06 = medium, 0.14 = large effect size) (28). A *p*-value ≤ 0.05 was considered statistically significant, and all tests were two-tailed. All *p*-values were reported accurately, with *p* < 0.001 used instead of *p* = 0.000.

## Results

Table 1 reveals that more than half (56.9%) of the studied women were in the age group of <45–<50 years old, with a mean age of 49.04 ± 3.49 years. Moreover, less than three-quarters of them (70.7%) had menopause less than 5 years ago. Concerning residence, more than half (51.7%) lived in rural areas. Furthermore, less than half (48.3%) had a university education. Regarding monthly income, more than two-thirds (67.2%) had insufficient income.

**Table 1:** Characteristics of the studied menopausal women (*n* = 58).

Personal characteristics	No	%
Age (in years)		
45–<50	33	56.9
50–55	25	43.1
Mean ± SD = 49.04 ± 3.49		
Duration since menopause		
Less than 5 years ago	41	70.7
5 or more years	17	29.3
Residence		
Rural	30	51.7
Urban	28	48.3
Level of education		
Primary education	4	6.9
Secondary or technical education	26	44.8
University education	28	48.3
Monthly income		
Enough	19	32.8
Not enough	39	67.2

Table 2 shows that there was a statistically significant difference among mean scores regarding menopausal women’s intrinsic and extrinsic environmental motivation at the pre-intervention, post-intervention, and follow-up phases (*p* < 0.001). The total mean score of intrinsic environmental motivation of the studied menopausal women was elevated from 12.59 ± 3.55 to 15.22 ± 2.79 and 15.67 ± 2.76; simultaneously, the total mean score of extrinsic environmental motivation of them was elevated from 7.72 ± 2.35 to 9.79 ± 1.90 and 10.22 ± 1.77 throughout intervention phases in favor of post-intervention and follow-up phases.

**Table 2:** Distribution of the studied women regarding their environmental motivation throughout the intervention phases (*n* = 58).

Environmental motivation factors	Pre-intervention	Post-intervention	Follow up	*F*	*p*-value	η^2^p (effect size)
	Mean ± SD	Mean ± SD	Mean ± SD			
Intrinsic motivation	12.59 ± 3.55	15.22 ± 2.79	15.67 ± 2.76	3.08	0.031	0.051
Extrinsic motivation	7.72 ± 2.35	9.79 ± 1.90	10.22 ± 1.77	2.59	0.038	0.043

Repeated measures analysis of variance (ANOVA) was used to compare changes across the three assessment points (pre-intervention, post-intervention, and follow-up). Partial eta squared (η^2^p) was used to estimate effect size.

**Figure 1:**
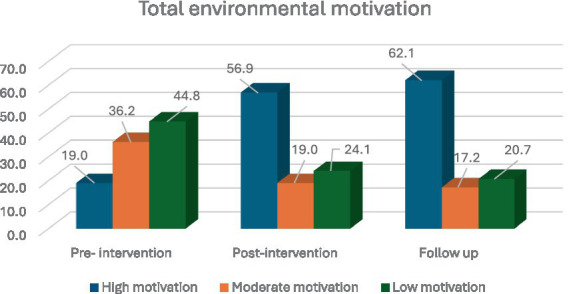
Percentage distribution of the studied menopausal women regarding total environmental motivation throughout the intervention phases (*n* = 58).

Figure 1 displays that 19.0, 56.9, and 62.1% of the studied menopausal women had high environmental motivation in the pre-intervention, post-intervention, and follow-up phases, respectively.

Table 3 indicates that there was a statistically significant difference among mean scores regarding menopausal women’s climate change health protection behavior factors at pre-intervention, post-intervention, and follow-up phases (*p* < 0.001). The total mean score of health protection behaviors related to climate change among menopausal women increased from 77.01 ± 6.56 to 92.17 ± 6.19 and 94.74 ± 5.62 throughout the intervention phases, in favor of the post-intervention and follow-up phases.

**Table 3:** Mean scores of the studied women’s behaviors for protecting health against climate change throughout the intervention phases (*n* = 58).

	Pre-intervention	Post-intervention	Follow up	ANOVA		η^2^p (effect size)
	Mean ± SD	Mean ± SD	Mean ± SD	*F*	*p*-value	
Behaviors related to the right information to protect health from global climate change	13.17 ± 3.37	15.98 ± 2.65	16.64 ± 2.41	24.27	< 0.001	0.299
Behaviors when encountering situations related to climate change	30.98 ± 4.47	35.33 ± 3.77	35.90 ± 3.27	27.95	< 0.001	0.329
Behaviors related to events that occur after climate change have passed.	17.66 ± 4.34	21.28 ± 3.40	22.03 ± 3.14	23.61	< 0.001	0.293
Behaviors to mitigate climate change	15.21 ± 2.46	19.59 ± 2.46	20.17 ± 2.07	77.86	< 0.001	0.577
Total score	77.01 ± 6.56	92.17 ± 6.19	94.74 ± 5.62	141.05	< 0.001	0.712

Repeated measures analysis of variance (ANOVA) was used to compare changes across the three assessment points (pre-intervention, post-intervention, and follow-up). Partial eta squared (η^2^p) was used to estimate effect size.

**Figure 2:**
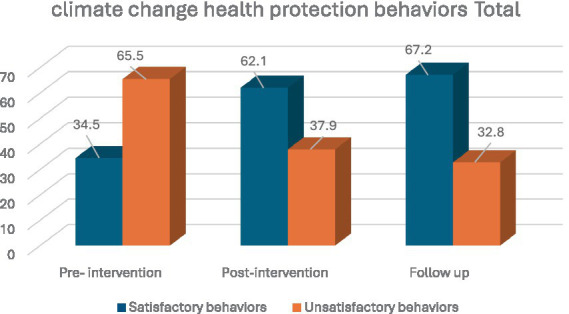
Percentage distribution of the studied women regarding their total climate change health protection behaviors throughout the intervention phases (*n* = 58).

Figure 2 shows that the proportion of menopausal women with satisfactory health protection behaviors related to environmental climate change increased over time, from 34.5% at the pre-intervention phase to 62.1% at the post-intervention phase and 67.2% at the follow-up phase, indicating sustained improvement across the study periods.

Table 4 indicates that there was a statistically significant difference among mean scores regarding menopausal women’s climate-sensitive health outcomes at the pre-intervention, post-intervention, and follow-up phases (*p* < 0.001). The total mean score of the climate-sensitive health outcomes of menopausal women decreased from 32.12 ± 7.68 to 25.00 ± 7.40 and 22.74 ± 7.12 throughout the intervention phases, in favor of the post-intervention and follow-up phases.

**Table 4:** Mean scores of studied women’s perceived climate-sensitive menopausal health outcomes throughout the intervention phases (*n* = 58).

Perceived climate-sensitive menopausal health outcomes	Pre-intervention	Post-intervention	Follow up	ANOVA		η^2^p (effect size)
	Mean ± SD	Mean ± SD	Mean ± SD	*F*	*p*-value	
Hot flushes	2.22 ± 0.91	1.86 ± 0.99	1.76 ± 0.96	3.76	0.026	0.062
Night sweats	1.28 ± 0.83	0.90 ± 0.81	0.79 ± 0.85	5.40	0.005	0.087
Heart palpitation	1.17 ± 0.79	0.84 ± 0.85	0.78 ± 0.85	3.71	0.027	0.061
Recurrent infections	1.38 ± 1.04	0.95 ± 0.86	0.84 ± 0.74	5.85	0.003	0.093
Sleeping disturbance	2.36 ± 1.11	1.97 ± 1.04	1.83 ± 1.07	3.82	0.024	0.063
Muscle and joint pain	2.29 ± 1.21	1.88 ± 1.18	1.69 ± 1.14	3.95	0.021	0.065
Lack of energy	2.29 ± 1.18	1.93 ± 1.15	1.76 ± 1.15	3.17	0.035	0.053
Physical discomfort and exhaustion	2.36 ± 1.11	2.02 ± 1.22	1.78 ± 1.18	3.64	0.029	0.060
Depressive mood	2.52 ± 0.88	2.07 ± 0.95	1.93 ± 0.95	6.29	0.002	0.099
Irritability	2.02 ± 1.20	1.67 ± 1.19	1.47 ± 1.18	3.15	0.041	0.052
Anxiety	1.78 ± 1.22	1.29 ± 1.21	1.17 ± 1.14	4.13	0.018	0.068
Stress	1.21 ± 0.16	1.21 ± 0.16	1.20 ± 0.15	0.12	0.720	0.002
Mood swings	1.90 ± 1.25	1.45 ± 1.15	1.33 ± 1.22	3.55	0.031	0.059
Lack of concentration	1.14 ± 0.92	0.76 ± 0.86	0.72 ± 0.91	3.75	0.028	0.062
Lack of sexual desire	1.98 ± 1.22	1.53 ± 1.15	1.41 ± 1.22	3.60	0.029	0.059
Impaired social relations	1.16 ± 0.98	0.86 ± 0.90	0.74 ± 0.71	3.41	0.032	0.056
Limited daily activities	1.40 ± 1.07	0.98 ± 1.11	0.93 ± 1.05	3.21	0.034	0.053
Decreased work productivity	1.05 ± 0.88	0.67 ± 0.82	0.59 ± 0.88	4.76	0.010	0.077
Total score	32.12 ± 7.68	25.00 ± 7.40	22.74 ± 7.12	25.32	<0.001	0.308

Repeated measures analysis of variance (ANOVA) was used to compare changes across the three assessment points (pre-intervention, post-intervention, and follow-up). Partial eta squared (η^2^p) was used to estimate effect size.

Table 5 clarifies that there was a statistically significant negative correlation between the total behavior score and total health outcome scores of the studied menopausal women at pre-intervention, post-intervention, and follow-up (*p* < 0.001).

**Table 5:** Correlation between the total behavior score and the total health outcome score of the studied women throughout the intervention phases (*n* = 58).

Variables	Total behaviors					
	Pre-intervention		Post-intervention		Follow up	
	*r*	*p*-value	*r*	*p*-value	*r*	*p*-value
Total health outcomes	−0.424	<0.001	−0.537	<0.001	−0.599	<0.001

Pearson correlation coefficients (*r*) with corresponding p-values. Correlation analysis was used to examine the relationship between total behaviors and total health outcomes.

## Discussion

This study utilized the Information–Motivation–Behavioral Skills (IMB) model to support menopausal women in managing perceived climate-related vulnerability in menopausal health outcomes. The study period revealed improvements in awareness, motivation, and behavioral skills. These findings are consistent with previous IMB-based research in environmental and public health contexts (23, 32, 36–39). Such patterns may reflect the structured nature of the IMB framework, which integrates information delivery, motivational enhancement, and behavioral skill development to facilitate health-related behavioral change over time rather than knowledge alone. To our knowledge, this study is among the first applications of the IMB model among menopausal women in a climate-related health context, suggesting potential relevance for culturally tailored health promotion strategies.

The findings demonstrated increases in both intrinsic and extrinsic environmental motivation among participants across the assessment phases, with a gradual rise in mean scores and an increased proportion of women classified as highly motivated from baseline to post-intervention and follow-up. These observed changes may be linked to the structured educational content of the intervention, which addressed both internal factors (such as perceived health relevance and self-care motivation) and external influences (such as social support and environmental responsibility). Orhan and Yağmur (40) reported similar patterns, observing improvements in health responsibility and stress management following motivational interventions in menopausal women. Likewise, Gibson et al. (41) reported that autonomy- and competence-supportive approaches enhance intrinsic motivation for pro-environmental behaviors. Dreijerink et al. (42) further emphasized the role of intrinsic and extrinsic motivation in sustaining environmental engagement, while Bakır et al. (43) highlighted improvements in knowledge, motivation, and behavioral skills following IMB-based interventions. Collectively, these findings are consistent with the IMB framework, which proposes that motivation is a key determinant of behavioral engagement.

Improvements were also observed in climate-related health protection behaviors among participants, with increased mean scores and a higher proportion of women reporting satisfactory behaviors across follow-up periods. These changes suggest a temporal association between participation in the intervention and adoption of adaptive behaviors. This pattern aligns with the theoretical premise of the IMB model, which emphasizes that behavioral skills are essential for translating knowledge and motivation into practice. However, given the absence of a control group, these findings should be interpreted as changes over time rather than confirmed intervention effects.

Similar findings have been reported in previous studies conducted in Egypt, where EL-Said et al. (44), Mohamed et al. (37), and Youssef et al. (45) documented improvements in adaptive health practices following structured educational interventions. Kurt et al. (32) also validated tools assessing climate protection behaviors and reported that structured education may enhance adaptive responses. Internationally, İşleyen and Kartal (46) and Lee and Park (47) found that IMB-based interventions were associated with improvements in preventive behaviors and self-management, while Park and Jang (48) demonstrated comparable outcomes using digital delivery approaches. Collectively, these studies support the relevance of IMB-based frameworks in promoting health-related behavioral engagement across different populations and settings.

Regarding perceived climate-sensitive menopausal health outcomes, the results indicated a reduction in symptom scores across the intervention phases, with mean values decreasing over time. Additionally, the proportion of women reporting better health outcomes increased from baseline to post-intervention and follow-up. These changes may reflect improved coping strategies and health behaviors developed during the intervention period. It is important to reiterate that the health outcomes assessed primarily represent classical menopausal symptoms. The term “climate-sensitive” was used to conceptualize these symptoms within a broader environmental stress context rather than to imply direct causation. Therefore, this study does not establish a causal relationship between climate change exposure and menopausal symptoms.

Supporting literature suggests that environmental conditions and climate factors may interact with menopausal symptom severity. Chauhan and Rahman (14) reported that women in warmer regions experience more severe symptoms, although lifestyle modifications may mitigate these effects. Sorensen et al. (49) highlighted that climate change may amplify gender-specific health vulnerabilities, particularly among postmenopausal women. Bala et al. (50) demonstrated that structured education improves health behaviors and reduces symptom burden, while Khandehroo et al. (51) found that health literacy interventions improve quality of life and adaptive capacity in menopausal women.

Overall, the observed patterns suggest that participation in the IMB-based educational program was associated with improvements in awareness, motivation, behaviors, and perceived health outcomes over time. However, due to the quasi-experimental one-group design, these findings should be interpreted cautiously, as causal inferences cannot be made and external or temporal factors may have contributed to the observed changes. This limitation calls for future controlled studies to confirm these associations and further evaluate the effectiveness of IMB-based interventions in similar populations.

As regards correlation findings, statistically significant associations were observed between environmental awareness, motivation, and behavioral scores across all phases (*p* ≤ 0.001). In addition, a significant negative correlation was found between behavioral scores and perceived health outcome scores (*p* ≤ 0.001). These relationships suggest that higher awareness and motivation are associated with greater engagement in protective behaviors, which in turn coincide with improved perceived health status. Previous studies have reported similar patterns. Galván-Mendoza et al. (52) demonstrated a link between environmental knowledge, perceived behavioral control, and green behavior. Yildirim et al. (53) found that environmental literacy influences behavioral engagement, while Castellini et al. (54) reported that health consciousness is associated with adoption of protective practices. These findings align with the IMB framework, which proposes that information and motivation influence outcomes primarily through behavioral skills (55). Overall, these results indicate consistent associations among psychosocial and behavioral variables; however, they do not confirm causality due to the study design limitations.

## Conclusion

Based on the methodological limitations of the study design, the findings suggest that the Information–Motivation–Behavioral Skills (IMB) model was associated with improvements in menopausal women’s awareness, motivation, and protective health behaviors, as well as a reduction in perceived climate change–sensitive health outcomes. Statistically significant differences were observed across the pre-intervention, post-intervention, and follow-up phases, in favor of the post-intervention and follow-up assessments, indicating sustained changes over time.

In addition, the results demonstrated statistically significant positive correlations between total environmental awareness and both motivation and behavioral scores across all study phases (*p* < 0.001). Conversely, statistically significant negative correlations were observed between total behavioral scores and total perceived health outcome scores across all phases (*p* < 0.001). These findings indicate that higher awareness and motivation were associated with improved behavioral practices and better perceived health outcomes, supporting the achievement of the study’s aim and the confirmation of the research hypotheses.

However, these findings should be interpreted with caution due to certain limitations, including the absence of a control group, which limits causal inference, and the reliance on self-reported measures, which may introduce reporting bias.

### Practical implications

The study suggests that the IMB model may be a useful framework for improving awareness, motivation, and protective behaviors among menopausal women, although findings should be interpreted as associative due to the study design.

In practice, the IMB model can be integrated into health education programs by nurses and other healthcare providers (physicians, midwives, and public health professionals) through structured education focused on information, motivation, and behavioral skills. Use of culturally tailored educational materials and inclusion of the IMB model in community and national health programs are recommended, especially in climate-vulnerable and rural settings. Collaboration between the health and environmental sectors and ongoing program evaluations are also encouraged.

For future research, larger and more diverse populations should be studied, with stronger designs such as randomized controlled trials and longer follow-up periods to confirm effectiveness. Comparative studies with other behavioral models and the use of digital health tools are recommended to enhance scalability. Further research is also needed in other vulnerable groups, along with training programs to improve healthcare providers’ ability to implement IMB-based interventions effectively.

### Limitations

The study has several limitations that should be considered when interpreting the findings. The use of purposive sampling from a single hospital administrative setting limits the generalizability of the results. In addition, the inclusion criteria restricted participation to married, literate women who had experienced natural menopause, which may have excluded groups potentially at higher risk of climate-related health vulnerabilities, such as women with lower socioeconomic status, limited education, rural residence, and higher environmental exposure. Therefore, the findings may not be representative of the broader population of menopausal women. Several potential confounding variables that may influence menopausal symptoms and health behaviors were not assessed or controlled for in this study, including body mass index, smoking status, physical activity level, hormone replacement therapy use, access to cooling or air-conditioning facilities, and socioeconomic status. Therefore, the observed changes cannot be attributed exclusively to the intervention.

## Data Availability

The original contributions presented in the study are included in the article/supplementary material, further inquiries can be directed to the corresponding author.
